# P-1984. Assessing the clinical impact of the BioFire FilmArray Blood Culture Identification 2 (BCID2) Panel in the Selection of Effective Antibiotic Therapy at an Academic Medical Center

**DOI:** 10.1093/ofid/ofaf695.2151

**Published:** 2026-01-11

**Authors:** Aamir S Dave, Stanley Moy

**Affiliations:** SUNY Downstate Health Sciences University, Hicksville, NY; SUNY Downstate Health Sciences University, Hicksville, NY

## Abstract

**Background:**

The BioFire FilmArray Blood Culture Identification (BCID2) panel uses polymerase chain reaction technology to rapidly identify pathogens and antimicrobial resistance genes in positive blood cultures. Using the BCID2 panel alongside an antimicrobial stewardship program has been proven to decrease time to effective targeted therapy. We aimed to evaluate the impact of the BCID2 panel in the management of bacteremia, focusing on the early administration of effective antibiotics.

**Figure:**
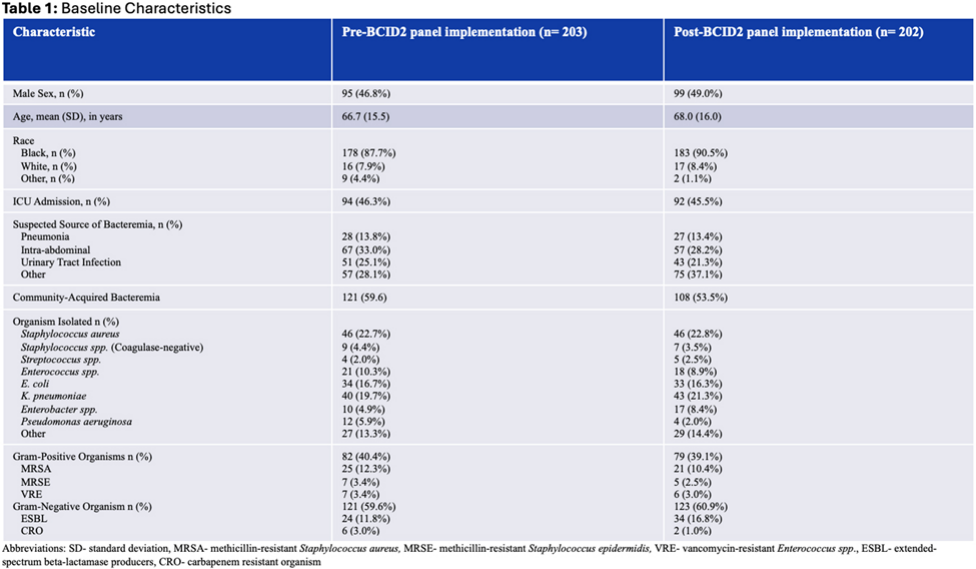

**Figure:**
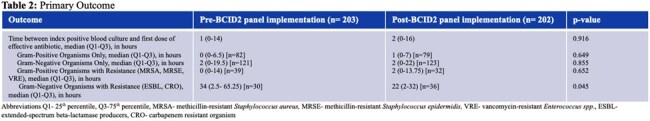

**Methods:**

Retrospective data was collected from adult patients with positive blood cultures at SUNY Downstate Health Sciences University between January 2021 to December 2024. The BCID2 panel was implemented in February 2022. The primary endpoint, of time between index positive blood culture and first dose of effective antibiotic, was assessed in participants before and after the implementation of the BCID2 panel. The secondary endpoints were time to appropriate antibiotic from index positive blood culture as defined by the institution’s BCID2 protocol, 30-day in-hospital mortality, and hospital length of stay.

**Figure:**
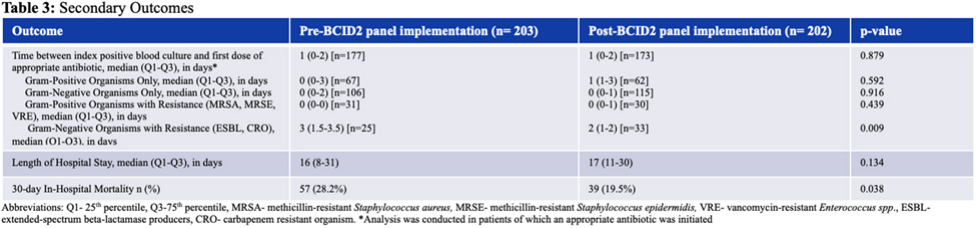

**Results:**

In total, 405 index positive blood cultures were sampled (n= 203 before the BCID2 panel implementation and n= 202 after). Baseline characteristics are presented in Table 1. The primary and secondary outcomes are summarized in Table 2 and 3, respectively. The median time between the index positive blood culture and first dose of effective antibiotic before and after BCID2 implementation was 1 vs. 2 hours, respectively (p= 0.916). For organisms with gram-negative resistance, the median time to effective antibiotics was 34 hours vs. 22 hours (p= 0.045).

**Conclusion:**

The BCID2 panel was not associated with shorter time between index positive blood culture and first dose of effective or appropriate antibiotic. When analyzing blood cultures that contained organisms with gram-negative resistance, the BCID2 panel was associated with a shorter time to effective and appropriate antibiotics.

**Disclosures:**

All Authors: No reported disclosures

